# Exploring the influence of situational interest on outdoor tourists’ hedonic and eudaimonic well-being

**DOI:** 10.3389/fpsyg.2025.1283929

**Published:** 2025-01-30

**Authors:** Peng Xu

**Affiliations:** School of Leisure Sports, Chengdu Sport University, Chengdu, China

**Keywords:** situational interest, hedonic well-being, eudaimonic well-being, time perception, autonomy need, outdoor tourism

## Abstract

**Introduction:**

This study introduces situational interest as a new factor influencing both hedonic and eudaimonic well-being in outdoor tourism settings. It explores how different dimensions of situational interest drive well-being and the mediation mechanisms involved.

**Methods:**

Data were collected from 642 respondents through an online self-report questionnaire on the Credamo platform. Structural equation modeling was used to analyze the relationships between situational interest dimensions, well-being outcomes, and mediating factors.

**Results:**

The results show that instant enjoyment directly enhances hedonic well-being. Novelty has a direct effect on hedonic well-being and an indirect effect on eudaimonic well-being. Attention demand influences hedonic well-being both directly and indirectly. Challenge and exploration intention indirectly promote eudaimonic well-being through the satisfaction of autonomy needs.

**Discussion:**

By uncovering the distinct pathways through which situational interest affects well-being, this study deepens our understanding of how outdoor tourism experiences can foster both immediate enjoyment and long-term personal growth. These findings provide practical insights for designing tourism activities that enhance tourists’ overall well-being.

## 1 Introduction

Well-being has long been a central pursuit in human life, discussed by philosophers for centuries and recognized as a core value by governments worldwide. In recent years, the focus on well-being has expanded to include a variety of domains, among which tourism has drawn increasing attention (Vada et al., 2020). Scholars note that travel experiences can contribute to both hedonic (pleasure-based) and eudaimonic (growth-oriented) well-being by encouraging enjoyment, personal growth, and positive psychological health. For example, visiting cultural heritage sites has been linked to existential authenticity and memorable experiences that increase subjective well-being (Yi et al., 2021). These findings underscore tourism’s potential role in enhancing people’s happiness and overall life satisfaction.

One point of view is that leisure, sports, and tourism, as the ways for people to escape from routine and tedious work and study, are the situations that scholars need to discuss to inspire people’s well-being (Mannell, 2007; Ahn et al., 2019). In outdoor tourism, researchers have shown that both tourism facilities—such as accommodations, food, and transportation—and personal attributes—such as tourists’ physical fitness and past travel experience—can affect well-being (Han et al., 2019; Korpela et al., 2014; Schlemmer et al., 2021). However, limited attention has been paid to the embodied nature of outdoor tourism (Humberstone, 2011), in which the mind and body work together to shape the travel experience. This embodied experience involves dynamic interactions between individuals and their surroundings. It can spark psychological states that influence well-being which yet remain underexplored in existing literature. Transformational tourism research further points to the importance of meaningful experiences that can lead to self-growth and overall societal benefits (Pope, 2018). Such perspectives suggest that deeper factors—beyond basic amenities—play a key role in boosting tourists’ happiness.

A potentially important yet understudied factor is situational interest. Defined as the temporary motivation or curiosity arising from specific contexts (Hidi and Anderson, 1992), situational interest differs from individual interest by being highly dependent on immediate circumstances (Hidi and Renninger, 2006). Within outdoor tourism, this concept is especially relevant because participants engage in activities that can trigger instant enjoyment, attention demand, novelty, challenge, and exploration. Although these dimensions may evolve throughout a trip, they collectively shape tourists’ hedonic and eudaimonic well-being in real time (Chen et al., 2006). Despite emerging research on well-being in various tourism settings, the influence of situational interest on different types of well-being has not been fully explored.

To address this gap, our study focuses on how the five dimensions of situational interest—instant enjoyment, attention demand, novelty, challenge, and exploration intention—relate to hedonic and eudaimonic well-being in the outdoor tourism context. By examining these relationships, we aim to clarify the processes through which situational interest shapes different aspects of well-being and shed light on potential mediation mechanisms. This study contributes to the literature on tourism and well-being by emphasizing the role of situational interest as a meaningful antecedent and offering a more detailed view of how tourism experiences can cultivate both immediate happiness and deeper personal growth.

From a practical standpoint, our findings may help outdoor tourism managers design activities that align with tourists’ changing interests. By offering experiences that engage participants physically and mentally, it may be possible to foster higher levels of well-being—both pleasure based and growth based. The subsequent sections outline the relevant literature, describe the methodology used to test our hypotheses, present the results of our analysis, and discuss the implications and future directions suggested by our findings.

## 2 Theoretical foundation

### 2.1 Hedonic and eudaimonic well-being

In recent years, the focus on well-being has expanded to include a variety of areas, with tourism receiving growing attention (Vada et al., 2020). Scholars note that travel experiences can promote both hedonic and eudaimonic well-being by fostering enjoyment, personal growth, and positive psychological health. For example, visiting cultural heritage sites can create existential authenticity and memorable experiences that increase subjective well-being (Yi et al., 2021). These findings highlight tourism’s potential to enhance life satisfaction and overall happiness. At the same time, tourism development can affect residents’ well-being in different ways, producing short-term challenges but leading to more positive long-term outcomes if managed sustainably (Godovykh et al., 2021). Moreover, the increasing use of information and communication technologies can enrich tourism experiences and improve well-being, but it also carries the risk of vacation disruptions that might reduce happiness (Gretzel and Stankov, 2021). These lines of research underscore the complex relationship between tourism and well-being, calling for further study of the factors that foster positive outcomes in various travel contexts.

In the broader literature, well-being is often discussed through concepts such as happiness, life satisfaction, and quality of life (Adger et al., 2022; Arola et al., 2023; Eger and Maridal, 2015). These terms generally refer to an individual’s sense of fulfillment in different areas of life. Two key orientations—hedonia and eudaimonia—have shaped much of the theoretical and empirical research on well-being. Rooted in the philosophical traditions of Democritus, hedonic well-being focuses on maximizing pleasure and minimizing pain (Giumetti and Kowalski, 2022). In contrast, Aristotle’s view of eudaimonic well-being centers on living in accordance with virtue, making rational choices, and seeking meaningful activities (Ryu et al., 2022; Sheth and Parvatiyar, 2022). While hedonic well-being emphasizes short-term pleasures, eudaimonic well-being involves deeper forms of personal development and purpose, cultivated over time.

In psychology, researchers increasingly view well-being as multidimensional, integrating both hedonic and eudaimonic perspectives (Ryff and Singer, 2008; Waterman, 2008). Subjective well-being, often associated with hedonia, typically includes measures of life satisfaction, positive emotions, and emotional balance (Chang et al., 2020; Diener et al., 2017; Disabato et al., 2016; Nowak-Olejnik et al., 2022). Eudaimonic well-being, meanwhile, is often assessed through scales that capture personal growth, autonomy, and purpose in life (Ryff and Keyes, 1995; Williams et al., 2022). Recent studies suggest that leisure pursuits, including tourism and outdoor activities, can promote both types of well-being. For instance, participation in eco-tourism has been linked to higher life satisfaction and a sense of purpose (Chen et al., 2022; Kim et al., 2020; Kono et al., 2020; Sirgy et al., 2017). Likewise, post-pandemic trends show that activities such as glamping provide enhanced nature connections and autonomy, which boost both hedonic enjoyment and eudaimonic fulfillment (Sun and Guo, 2022). Even virtual or remote tourism can offer hedonic pleasure and encourage deeper reflection when physical travel is limited (Stankov and Filimonau, 2023).

Together, these findings affirm that well-being in tourism contexts is multifaceted. Hedonic well-being emerges from pleasant and engaging experiences, while eudaimonic well-being develops through meaningful, growth-oriented activities. As research in this field continues to grow, further exploration of both pleasure-driven and purpose-driven aspects of tourism can help scholars and practitioners design experiences that maximize travelers’ overall well-being, whether through traditional leisure travel, immersive cultural experiences, or innovative virtual platforms.

### 2.2 Situational interest

Situational interest has been widely recognized in psychology and education as a key factor that shapes an individual’s engagement and motivation to learn (Chen and Wang, 2017; Roure and Lentillon-Kaestner, 2022). It generally refers to a psychological state of involvement or willingness to participate in a particular activity (Cheng et al., 2023). Hidi and Anderson (1992) were among the first to distinguish between two types of interest: situational and individual. Hidi (2001) emphasized that situational interest includes all interest triggered by the environment, while Wheatley et al. (2024) described it as an interactive response—one that arises from an individual’s reaction to a given context. Deci et al. (1991) further noted that this form of interest depends heavily on how a person perceives the inherent characteristics of the activity itself.

Recent research by Sun et al. (2023) characterizes situational interest as a brief surge of curiosity or motivation sparked by specific contexts, highlighting its tendency to shift based on the situation (Roure and Dieu, 2023). In a tourism setting, situational interest explains why travelers experience different motivations in varying environments. Needham and Szuster (2011), for example, investigated how ecological impact or overcrowding in tourist destinations influences visitors’ acceptance of management strategies, demonstrating that situational interest can drive responses to environmental changes in coastal regions.

The five dimensions of situational interest—instant enjoyment, attention demand, novelty, challenge, and exploration intention—stem from theoretical research across education, psychology, and tourism. Mitchell (1993) introduced a model that distinguished how interest is both generated and maintained, while Hidi and Harackiewicz (2000) emphasized the role of perceived relevance in these processes. Hidi and Renninger (2006) advanced this view by identifying four stages of interest development. Building on these foundations, Roure et al. (2016) proposed five specific dimensions that capture the multifaceted nature of situational interest. These dimensions are particularly useful in understanding outdoor tourism because they reflect the varied experiences tourists may encounter in natural or adventure-oriented settings (Roure and Lentillon-Kaestner, 2022).

In tourism research, situational interest helps explain how visitors engage with activities or destinations. Trauer (2006) discussed the importance of situational involvement in Special Interest Tourism (SIT), suggesting that factors such as novelty, challenge, and personal relevance influence tourists’ behavior. More recently, scholars have underscored the role of situational interest in supporting tourists’ well-being and engagement in post-pandemic contexts, where outdoor and leisure tourism options such as glamping have become increasingly popular (Lu et al., 2024). These studies point to how situational interest can promote deeper involvement in tourism experiences by highlighting the importance of environmental conditions and personal motivations.

Past research on situational interest has primarily focused on learning and performance outcomes. It has been linked to enhanced academic performance across subjects and settings, including higher education, sports, and technology-based learning environments (Kolovelonis and Goudas, 2023; Roure and Dieu, 2023; Roure and Lentillon-Kaestner, 2022; Sun et al., 2023). Furthermore, situational interest tends to increase engagement and motivation, leading to greater effort and persistence in tasks (Ainley, 2006). In the tourism domain, a similar pattern emerges, wherein situational interest can heighten travelers’ willingness to engage with destinations and activities, often due to factors such as novelty, perceived relevance, or emotional connection to the environment (Needham and Szuster, 2011). Cheng et al. (2023) also highlight how situational interest fosters positive attitudes in immersive settings, which parallels findings in tourism research suggesting that such interest contributes to positive, enriching experiences.

### 2.3 Hypothesis development

Outdoor tourism, shaped by situational interest, offers a distinctive leisure experience (Liu and Geng, 2023). The connection between the activity and an individual’s goals, such as personal development, further influences their interest (Harackiewicz and Hulleman, 2010). When individuals perceive alignment between outdoor tourism and their aspirations, it enhances their interest. According to situational interest theory, a profound personal connection to a specific location or activity, like a cherished family vacation spot or a beloved hiking trail, fosters interest, leading to various positive outcomes (Knogler et al., 2015).

**Figure 1:**
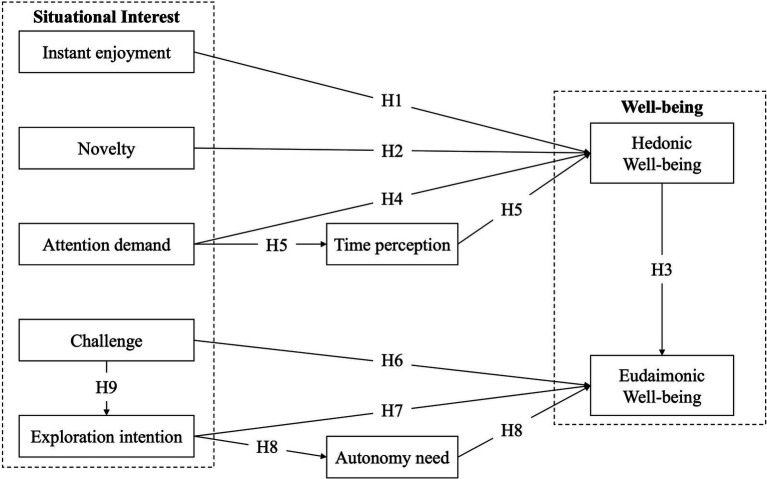
Conceptual model.

Engaging in activities that resonate with personal interests often translates into heightened feelings of enjoyment and satisfaction, contributing to hedonic well-being (Kim et al., 2020). In addition, individuals tend to seek and absorb information about topics that interest them, enriching their knowledge and understanding and contributing to their overall well-being (Fan and Luo, 2022). Thus, we propose examining the mechanisms between the five dimensions of situational interest and two categories of well-being; the conceptual model is shown in Figure 1.

*H1: The instant enjoyment of situational interest has a significant positive influence on outdoor tourists’ hedonic well-being.*

Moreover, emerging evidence supports the notion that the novelty of outdoor tourist experiences correlates positively with hedonic well-being. Novel experiences are often more enjoyable, fostering greater feelings of pleasure and happiness (Bagheri and Milyavskaya, 2020). Recent studies emphasize that novel experiences contribute substantially to tourists’ overall satisfaction and happiness during outdoor activities (Blomstervik and Olsen, 2022). Thus, we hypothesize as follows:

*H2: The novelty of situational interest has a significant positive influence on outdoor tourists’ hedonic well-being.*

We propose hedonic well-being as an explanatory mechanism between novelty and eudaimonic well-being. The inherent human curiosity driving the pursuit of novel experiences is considered a crucial element of well-being (Kashdan and Silvia, 2009; Su et al., 2023). Individuals engaging in new and unfamiliar outdoor activities often report a heightened sense of self-discovery and personal growth, contributing significantly to eudaimonic well-being (Waterman and Schwartz, 2024). Steger and Kashdan’s (2013) research suggests that individuals highly involved in novelty activities experience greater hedonic well-being, which, as an integral element, contributes to the development of meaning in life. Studies propose that satisfaction with novelty predicts hedonic well-being and subsequently motivates personal development, thereby influencing eudaimonic well-being (Al-okaily et al., 2023; González-Cutre et al., 2016). Thus, we hypothesize as follows:

*H3: Hedonic well-being mediates the relationship between the novelty of situational interest and eudaimonic well-being.*

Outdoor tourism, requiring tourists’ attention, fosters an increased sense of connection to nature and provides opportunities for activities that promote mindfulness and relaxation (Farkic, 2021). Flow experiences, often characterized as enjoyable, satisfying, and beneficial for both physical and mental well-being, are commonly associated with concentrated attention (Ilies et al., 2017). Tourists engaged in outdoor activities requiring focused attention are likely to reach a flow state, often reporting feelings of happiness, satisfaction, and fulfillment. This is likely to have a positive impact on a person’s hedonic well-being, encompassing their overall sense of pleasure and satisfaction in life. Thus, we hypothesize as follows:

*H4: The attention demand of situational interest has a significant positive influence on outdoor tourists’ hedonic well-being.*

Existing literature strongly supports the relationship between attention demand and time perception (Block and Gruber, 2014; Livesey et al., 2007). Matthews and Meck’s (2016) systematic review elucidates the critical role of attention in time perception, detailing how subjective duration is influenced by both non-temporal stimulus properties and the allocation of processing resources. Meta-analysis studies further emphasize that task attention demand and attention processes are the determining factors in the outcome of time perception (Zheng et al., 2022). Flow theory posits that engaging in challenging activities with a suitable attention demand can result in an expanded sense of time, linked with feelings of enjoyment and satisfaction (Boniface, 2000). Thus, we hypothesize as follows:

*H5: The time perception mediates the relationship between attention demand and hedonic well-being of outdoor tourists.*

A sense of purpose or meaning in one’s activities and interests has consistently demonstrated associations with elevated levels of hedonic and eudaimonic well-being, providing individuals with a profound sense of direction and fulfillment (Pritchard et al., 2020). Recent studies in outdoor tourism have further elucidated that this form of recreation triggers the challenge and exploration intentions of tourists, presenting unique opportunities for personal growth, learning, and self-discovery. This, in turn, contributes to a more profound sense of purpose and meaning in life (Williams and Soutar, 2009). Ritpanitchajchaval et al. (2023) delved into the motives propelling mountain-based adventure tourism and its impact on the development of eudaimonic well-being, suggesting a positive relationship between engaging in challenging outdoor activities and the elevation of eudaimonic well-being. Moreover, Pomfret et al. (2023) conducted a systematic literature review, and the findings from this review indicate a conceptual link between engaging in outdoor adventure activities and well-being. Drawing from this evidence, we propose that tourists’ exploration intentions during outdoor tourism significantly contribute to their eudaimonic well-being. Thus, we hypothesize as follows:

*H6: The challenge of outdoor tourists has a significant positive influence on their eudaimonic well-being.*

*H7: The exploration intention of outdoor tourists has a significant positive influence on their eudaimonic well-being.*

In this study, we introduce autonomy need as a potential explanatory factor in the relationship between exploration intention and eudaimonic well-being. Grounded in Self-Determination Theory (SDT), which posits that the intrinsic psychological need for autonomy is crucial for human well-being and optimal functioning (Karbo et al., 2024), SDT suggests that when the need for autonomy is fulfilled, individuals are more likely to engage in activities aligning with their personal goals and interests, consequently experiencing eudaimonic well-being and happiness (Lee et al., 2022). Exploration intention, as a form of self-need, reflects the desire to seek out new experiences, information, and perspectives. It is conceptualized as a facet of autonomy need, driven by an individual’s internal motivation to learn, grow, and expand their understanding of the world (Kouali et al., 2022). Based on this conceptualization, we propose the following:

*H8: The autonomy need mediates the relationship between exploration intention and eudaimonic well-being of outdoor tourists.*

Building on recent literature, we find that overcoming challenges in outdoor activities can indeed contribute to a sense of personal development, fostering an increased inclination toward exploration intention (Hoffner and Bond, 2022). Research by Mulyadi et al. (2016) supports this, suggesting that individuals, upon overcoming challenges, often experience a heightened sense of accomplishment and pride, leading to elevated self-esteem and self-efficacy. Thus, we hypothesize as follows:

*H9: The exploration intention and autonomy need order mediate the relationship between challenge and eudaimonic well-being of outdoor tourists.*

## 3 Methodology

### 3.1 Data collection and participants

This study aimed to examine how situational interest influences outdoor tourists’ well-being. Data were collected in October 2022 using Credamo, an online platform widely employed by social science researchers for survey-based studies (Gong et al., 2020). Although Credamo does not represent all outdoor tourists, its user pool spans diverse demographic backgrounds, making it a suitable starting point for research on tourism-related behaviors. We used a screening process to ensure participants had actual outdoor tourism experience. Potential respondents were first asked, “Have you ever participated in outdoor tourism?” Only those who answered “yes” were allowed to proceed with the survey, which measured situational interest (e.g., instant enjoyment), related mediating variables (e.g., time perception), well-being outcomes (i.e., hedonic well-being), and demographic information.

To encourage participation, a small incentive (USD 0.25) was offered to each respondent. A total of 693 individuals completed the questionnaire, and after removing invalid or incomplete responses, 642 valid samples remained. Most participants were between 21 and 35 years old (75.2%), 38.3% were female, and 78.8% held a 4-year university degree or a master’s degree.

### 3.2 Measurement items

#### 3.2.1 Situational interest

Following Roure et al. (2016), situational interest in the context of outdoor tourism experiences, we employed subjective measures derived from the conceptualization and operationalization detailed in the research. The Roure Situational Interest Scale (RSIS) serves as an instrument for assessing an individual’s interest level in a specific activity, contingent upon the context or situation in which it unfolds. The RSIS 2016 is a self-report scale comprising 15 items, each rated on a 5-point Likert scale, ranging from “not at all” to “extremely.” These items gauge an individual’s degree of instant enjoyment, attention demand, novelty, challenge, and exploration intention within a given activity (see Table 1). The scale has demonstrated robust reliability and validity across various samples, encompassing children, adolescents, and adults.

**Table 1:** Measurement items and sources.

Items	Sources
**Instant enjoyment (IE)**	Roure et al. (2016)
IE 1 “What I am doing was appealing to me”	
IE 2 “What I did was enjoyable for me”	
IE 3 “What I am doing inspires me to try out what I am doing”	
**Attention demand (AD)**	
AD1 “What I am doing demanded my high attention”	
AD2 “I was focused on what I am doing today”	
AD3 “I was concentrated on what I am doing”	
**Novelty (NV)**	
NV1 “What I am doing was a new activity for me to do”	
NV2 “What I am doing today was fresh/new”	
NV3 “What we did today was new to me”	
**Challenge (CL)**	
CL1 “What I am doing was complex”	
CL2 “What I am doing was complicated”	
CL3 “What I am doing was hard for me to do”	
**Exploration intention (EI)**	
EI1 “I wanted to know more about how to do what I am doing today”	
EI2 “I wanted to analyze and have a better handle on what I am doing today”	
EI3 “I’d like to know more about how to do what I am doing”	
**Time perception (TP)**	Rudd et al. (2012)
TP1 “I have lots of time in which I can get things done”	
TP2 “Time is slipping away” (reverse-scored)	
TP3 “Time is expanded”	
TP4 “Time is boundless”	
**Autonomy need (AN)**	Gagné (2003)
AN1 “When doing this activity, I feel free to be who I am”	
AN2 “I have a say in what happens and can voice my opinion”	
AN3 “I feel controlled and pressured to be certain ways” (reverse-scored)	
**Hedonic well-being (HW)**	Diener et al. (2017)
HW1 “I felt happy during the trip”	
HW2 “I felt excited during the trip”	
HW3 “After the trip, I was in good health and spirits”	
**Eudaimonic well-being (EW)**	Ryff and Keyes (1995)
EW1 “This trip has improved my perspective”	
EW2 “This trip has taught me to be more optimistic about life”	
EW3 “This trip keeps me thinking positively about myself and the world”	
EW4 “This trip gave me a lot of life insights”	
EW5 “This trip made me feel that I have grown”	

#### 3.2.2 Time perception

To assess time perception, this study adopts the definition provided by Rudd et al. (2012), wherein time perception is characterized as the degree to which individuals subjectively experience the duration of events and the temporal ordering of those events. In essence, time perception is not merely a passive mechanism for gauging the passage of objective time but an active process that encompasses the interpretation and organization of sensory information. The measurement items were referenced, with responses rated on a 5-point scale (1 = *not at all*, 5 = *strongly agree*). Consequently, a 4-item time perception scale was employed (see Table 1).

#### 3.2.3 Autonomy need

In psychological literature, basic psychological needs are understood as fundamental requirements that must be fulfilled for individuals to optimize their functioning (Karbo et al., 2024). One of these needs is the need for autonomy, which pertains to the desire for self-direction and control over one’s own life. Various methods have been employed to measure autonomy need, with one common approach being based on the General Needs Satisfaction Scale (GNSS) developed by Gagné (2003). In this study, according to GNSS, autonomy need was measured using three self-reported items (1 = *not at all*, 5 = *strongly agree*).

#### 3.2.4 Hedonic well-being

It is the subjective experience of one’s emotions and moods and can be thought of as the “hedonic tone” of one’s life. Hedonic well-being includes positive emotions and satisfaction, as well as the absence of negative emotions such as sadness, anger, and anxiety. The measurement of hedonic well-being primarily relies on the Subjective Happiness Scale (SHS). Subjective well-being is commonly divided into two components: cognition and emotion, comprising positive emotion and life satisfaction (Diener et al., 2017). The measurement items drawn from the SHS were consulted, a 3-item hedonic well-being was presented based on a 5-point Likert-type scale with “*not at all*” to “*strongly agree*” anchored statements (see Table 1).

#### 3.2.5 Eudaimonic well-being

In literature, eudaimonic well-being refers to the sense of meaning and purpose in one’s life, and the feeling that one’s life is going in a direction that is consistent with one’s values and beliefs. Ryff and Keyes (1995) according to self-determination theory developed a multidimensional measure of psychological well-being (PWB). It is considered a robust measure of eudaimonic well-being and has been widely used. In this study, eudaimonic well-being was measured by a 5-item scale from PWB (1 = *not at all*, 5 = *strongly agree*).

### 3.3 Data analysis

The data analysis consists of two steps. To assess the measures given in the data in this study context, the first step was to test the adequacy of the measurement model with a confirmatory factor analysis (CFA) using IBM SPSS 24.0 and AMOS version 22. Several criteria were used to assess the model fit (i.e., Cronbach’s alpha). Next, to test the hypotheses, the second step was to assess the adequacy of the structural model using the AMOS program. The analysis of mediation was performed using the PROCESS 4.0 program in SPSS. Based on bootstrapping with 5,000 bootstrap resamples, appropriate parameter estimate was selected to examine indirect effects in mediation models.

## 4 Results

### 4.1 Reliability and validity analysis

The measurement model of nine variables (i.e., instant enjoyment) was analyzed by conducting convergent validity, discriminant validity, and reliability analysis. The results suggest that the measurement model of this study is adequate. As presented in Table 2, all Kaiser–Meyer–Olkin (KMO) values are above the cutoff point of 0.5, implying that the data are adequate for factor analysis (Fuller et al., 2016). All Cronbach’s alpha (*α*) reported acceptable values, and all the survey item loadings are above 0.6. Meanwhile, the average variance extracted (AVE) values of all latent variables are above the cutoff point of 0.5, implying an adequate convergent validity (Kleijnen et al., 2007). The composite reliability (CR) values of all latent variables ranged between 0.885 and 0.958 and are above the cutoff point of 0.7. Furthermore, discriminant validity was assessed by comparing the values of square roots of AVE of all latent variables that are larger than the correlations between variables. As shown in Table 3, this indicates that discriminant validity is supported. Furthermore, we applied the maximum-likelihood estimation method to verify the factorial validity of the scale. The fit indices are above the threshold of 0.9: Comparative Fit Index (CFI) = 0.921 and Tucker-Lewis Index (TLI) = 0.936. The value of Root Mean Square Error of Approximation (RMSEA) = 0.065 indicates a good model fit, and the value of Standardized Root Mean Square Residual (SRMR) = 0.056 is below the threshold of 0.09. Therefore, these criteria suggest the measurement model acceptable.

**Table 2:** Confirmatory factor analysis.

Items	λ	α	KMO	CR	AVE
**Instant enjoyment (IE)**		0.887	0.754	0. 904	0.759
IE 1	0.859				
IE 2	0.882				
IE 3	0.873				
**Attention demand (AD)**		0.904	0.827	0.926	0.806
AD1	0.915				
AD2	0.912				
AD3	0.865				
**Novelty (NV)**		0.777	0.766	0.837	0.631
NV1	0.824				
NV2	0.759				
NV3	0.798				
**Challenge (CL)**		0.877	0.809	0.904	0.759
CL1	0.869				
CL2	0.863				
CL3	0.881				
**Exploration intention (EI)**		0.869	0.773	0.885	0.720
EI1	0.851				
EI2	0.839				
EI3	0.855				
**Time perception (TP)**		0.899	0.752	0.952	0.831
TP1	0.917				
TP2	0.914				
TP3	0.904				
TP4	0.911				
**Autonomy need (AN)**		0.868	0.718	0.919	0.791
AN1	0.863				
AN2	0.918				
AN3	0.886				
**Hedonic well-being (HW)**		0.837	0.726	0.902	0.754
HW1	0.871				
HW2	0.872				
HW3	0.862				
**Eudaimonic well-being (EW)**		0.903	0.740	0.958	0.819
EW1	0.932				
EW2	0.854				
EW3	0.901				
EW4	0.923				
EW5	0.914				

λ, factor loading; α, Cronbach’s alpha; KMO, Kaiser–Meyer–Olkin; CR, composite reliability; AVE, average variance extracted.

**Table 3:** Discriminant validity.

Variables	1	2	3	4	5	6	7	8	9
1. IE	**0.865**								
2. AD	0.500	**0.881**							
3. NV	0.269	0.314	**0.775**						
4. CL	0.501	0.488	0.179	**0.855**					
5. EI	0.577	0.598	0.311	0.636	**0.847**				
6. TP	0.569	0.637	0.289	0.501	0.636	**0.912**			
7. AN	0.584	0.582	0.256	0.422	0.442	0.725	**0.890**		
8. HW	0.570	0.653	0.313	0.497	0.599	0.614	0.602	**0.868**	
9. EW	0.404	0.516	0.180	0.521	0.398	0.354	0.399	0.517	**0.984**

Square roots of AVE values in the diagonal as bold. IE, instant enjoyment; NV, novelty; AD, attention demand; CL, challenge; EI, exploration intention; TP, time perception; AN, autonomy need; HW, hedonic well-being; EW, eudaimonic well-being.

### 4.2 Common method bias testing

The common method bias refers to the phenomenon in which a survey is influenced by the methods used to collect the data. This bias can occur when a single method, in this study self-report, is used to measure multiple constructs; there may be a common method bias problem (Podsakoff and Organ, 1986). Therefore, the Harman single factor test is used to test the common method bias in the SPSS program. The results show that there is no common method bias in the data. The variance interpretation rate of the first factor is 20.31%, accounting for 26% of the total variance interpretation rate (78.21%), which is less than 50% when the first factor is not rotated (Fuller et al., 2016).

### 4.3 Hypothesis estimation

**Figure 2:**
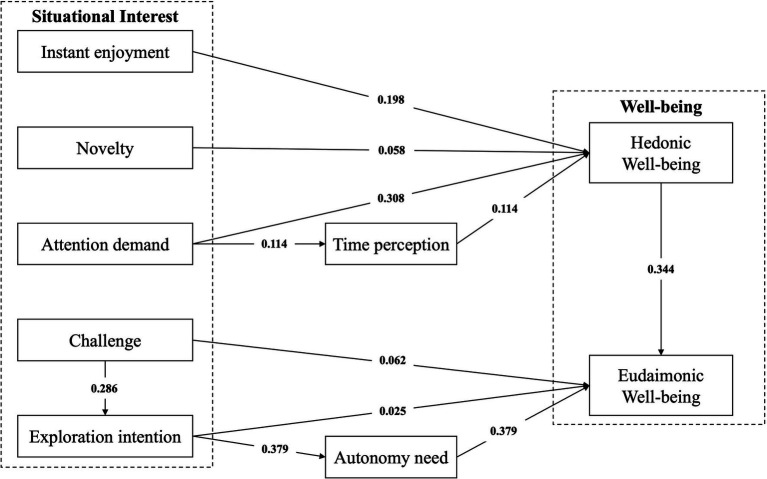
Results of hypothesis.

The SEM approach using AMOS 22.0 was performed to examine the relationship between the variables, and the PROCESS 4.0 program in SPSS was consulted to estimate the mediation effect in the research framework. As presented in Table 4, instant enjoyment has a significant positive effect on hedonic well-being of outdoor tourists (*β =* 0.198; *t* = 3.056; *p* = 0.000), providing support for H1. Both novelty and attention demand have a significant positive effect on hedonic well-being of outdoor tourists (*β =* 0.058, *t* = 1.812, *p* = 0.032; *β =* 0.308, *t* = 4.042, *p* = 0.000), supporting H2 and H4. However, it can be observed that both challenge and exploration intention have no significant positive effect on eudaimonic well-being (*β =* 0.062, *t* = 0.481, *p* = 0.427; *β =* 0.025, *t* = 0.104, *p* = 0.197), not supporting H6 and H7. As hypothesized, hedonic well-being mediates the relationship between novelty and eudaimonic well-being (*β =* 0.344; *t* = 13.182; *p* = 0.000), providing support for H3. The results also show that time perception partially mediates the relationship between attention demand and hedonic well-being (*β =* 0.114; *t* = 10.334; *p* = 0.000), supporting H5. Furthermore, autonomy need completely mediates the relationship between exploration intention and eudaimonic well-being (*β =* 0.379; *t* = 28.565; *p* = 0.000), supporting H8. Finally, exploration intention and autonomy need order mediate the relationship between challenge and eudaimonic well-being (*β =* 0.286; *t* = 18.213; *p* = 0.000), supporting H9 (see Figure 2).

**Table 4:** Hypotheses testing results.

Hypotheses	Path	*β*	*t-*value	*p-*value	Result
Direct effects					
H1	IE → HW	0.198	3.056	0.000	Supported
H2	NV → HW	0.058	1.812	0.032	Supported
H4	AD → HW	0.308	4.042	0.000	Supported
H6	CL → EW	0.062	0.481	0.427	Rejected
H7	EI → EW	0.025	0.104	0.197	Rejected
Indirect effects					
H3	NV → HW → EW	0.344	13.182	0.000	Supported
H5	AD → TP → HW	0.114	10.334	0.000	Supported
H8	EI → AN → EW	0.379	28.565	0.000	Supported
H9	CL → EI → AN → EW	0.286	18.213	0.000	Supported

IE, instant enjoyment; NV, novelty; AD, attention demand; CL, challenge; EI, exploration intention; TP, time perception; AN, autonomy need; HW, hedonic well-being; EW, eudaimonic well-being.

## 5 Discussion

This study examined how five dimensions of situational interest—instant enjoyment, novelty, attention demand, challenge, and exploration intention—relate to hedonic and eudaimonic well-being in an outdoor tourism setting. Overall, the findings suggest that the majority of the hypothesized relationships hold true, with two notable exceptions (H6 and H7).

The results confirm that instant enjoyment, novelty, and attention demand significantly foster hedonic well-being among outdoor tourists, aligning with prior studies indicating that enjoyable or novel experiences can enhance positive affect (Bagheri and Milyavskaya, 2020; Blomstervik and Olsen, 2022). Emphasizing attention demand is consistent with the perspective that activities requiring deep engagement can produce flow-like states that boost immediate pleasure (Ilies et al., 2017). Moreover, time perception was found to mediate the effect of attention demand on hedonic well-being, suggesting that losing awareness of time when fully immersed intensifies short-term happiness. Furthermore, novelty’s indirect impact on eudaimonic well-being through hedonic well-being supports Steger and Kashdan’s (2013) view that initial enjoyment may pave the way for deeper personal meaning.

Despite these findings, challenge and exploration intention did not exhibit the expected direct effects on eudaimonic well-being. While previous research underscores the transformative potential of challenging outdoor activities (Ritpanitchajchaval et al., 2023; Pomfret et al., 2023), our data indicate that challenge more effectively promotes growth when it operates through exploration intention and autonomy need, rather than by itself. A possible explanation is that for some participants, highly demanding tasks can trigger stress or self-doubt, undermining the likelihood of meaningful growth. Another possibility is a “threshold” effect: Moderate challenges may inspire a sense of achievement, but if the demands surpass perceived skill levels, anxiety or fatigue might overshadow the potential for deeper fulfillment.

Similarly, exploration intention alone did not generate eudaimonic well-being but required the fulfillment of autonomy need—an idea that aligns with Mulyadi et al.’s (2016) emphasis on individual agency. Simply wanting to discover new experiences may not suffice if tourists feel they lack the freedom or capacity to act on that desire. In such situations, exploration risks become either unstructured or obligatory, limiting opportunities for meaningful reflection and a sustained sense of personal value.

Taken together, these results highlight the nuanced ways in which situational interest shapes well-being in outdoor tourism. While instant enjoyment, novelty, and attention demand directly enhance hedonic pleasure, eudaimonic well-being appears more context-dependent. The non-significant findings for H6 and H7 suggest that challenging activities and exploration intentions do not inherently produce deeper fulfillment. Instead, their benefits emerge through mediating factors such as time perception and autonomy need, which enable tourists to internalize and learn from their experiences. This complexity underscores the importance of examining both immediate affective rewards and more enduring psychological processes when considering how situational interest supports tourists’ well-being.

## 6 Theoretical implications

Previous research has consistently shown that well-being in tourism settings is influenced by various factors, including visitors’ personal traits, environment-related elements, and interactive experiences (Ahn et al., 2019; Schlemmer et al., 2021). Building on this scholarship in the context of sports tourism, the present study examined how specific dimensions of situational interest—namely, instant enjoyment, novelty, attention demand, challenge, and exploration intention—foster both hedonic and eudaimonic well-being. The findings indicate the importance of these dimensions, suggesting that sports tourism settings can provide “live” contexts in which psychological states and physical engagement can reinforce each other, ultimately enhancing visitors’ overall well-being.

Consistent with earlier research on outdoor activities (Han et al., 2019; Yi et al., 2021), our results indicate that instant enjoyment, attention demand, and novelty are especially strong predictors of hedonic well-being. Sports tourists, who often seek excitement and new experiences, tend to experience immediate benefits when engaging in stimulating and enjoyable activities—whether it is the thrill of unfamiliar tasks or the focused immersion demanded by competitive or physically challenging pursuits. This aligns with the research on flow states (Hancock et al., 2019), where complete attention in a novel setting leads to positive affect. Meanwhile, challenge and exploration intention show more complex relationships with eudaimonic well-being; their positive effects emerge more clearly under conditions such as adequate skill levels or a sense of competence. These findings resonate with studies suggesting that excessive difficulty or unstructured exploration may lead to anxiety or stress, thereby diminishing eudaimonic gains (Bagheri and Milyavskaya, 2020).

By uncovering these nuanced patterns, this study adds depth to the understanding of situational interest as a multidimensional construct. Unlike one-dimensional views that focus solely on short-term pleasure in tourism, the current evidence suggests that tourists’ drive to explore, overcome challenges, and immerse themselves in new environments can yield both immediate emotional rewards and longer-term growth. This duality aligns with prior discussions of hedonic versus eudaimonic outcomes (Vada et al., 2020), emphasizing that sports tourism can promote not only momentary excitement but also personal development. Furthermore, the partial mediating pathways observed—such as the role of novelty in connecting instant enjoyment to deeper psychological fulfillment—clarify how immediate pleasure and exploration may evolve during a sports-related trip. These points also support transformational tourism theories (Pope, 2018), indicating that engagement with challenging or unfamiliar conditions can inspire self-reflection, autonomy, and purpose.

Taken together, the study extends theoretical discussions by demonstrating that the embodied, context-dependent features of sports tourism are integral to well-being. By incorporating concepts such as flow, self-determination theory, and the multidimensional structure of situational interest, the study offers a more precise view of how visitors’ psychological processes unfold. These findings address the identified gaps in research on the drivers of well-being in sports tourism (Korpela et al., 2014; Humberstone, 2011), providing new evidence that deeper emotional or cognitive engagement—rather than physical facilities alone—is essential for generating long-lasting benefits. As such, the results build on prior research that highlights the dynamic interaction between tourists and their environments, particularly when these environments are physically challenging, novel, or inherently motivating.

## 7 Managerial implications

From a practical standpoint, these findings offer multiple insights for sports tourism operators, event organizers, and policymakers aiming to enhance tourists’ hedonic enjoyment and eudaimonic growth. First, emphasizing instant enjoyment can boost overall satisfaction and draw a wider audience. Providing accessible yet appealing physical activities (e.g., beginner-friendly adventure courses or scenic running trails) can fulfill tourists’ desire for immediate fun while still presenting appropriate challenges. This aligns with flow theories that recommend matching skill levels with task difficulty to sustain engagement.

Second, novelty—expressed through innovative programming or unique local experiences—can serve as a critical differentiator. For sports tourism destinations that compete on an international scale, designing events or venues that highlight distinctive cultural or ecological attributes can amplify participants’ sense of wonder. In doing so, managers can help convert initial curiosity into both immediate excitement and subsequent feelings of achievement.

Third, attention demand appears to be an important factor in enhancing hedonic well-being. Incorporating interactive technologies, such as augmented reality (AR) for race tracking or virtual reality (VR) for simulated training, can increase engagement and immersion. These methods have practical relevance in a digital age where tourists often expect technology-enhanced experiences.

Finally, sports tourism planners should recognize that while challenge and exploration can sometimes have ambiguous outcomes, with proper support they can facilitate deeper personal transformation. Structuring programs that include goal-setting workshops, dividing participants by skill level, and providing reflective debriefing sessions can help visitors process challenging tasks more productively. Likewise, mentorship from local guides or experienced athletes can foster learning and encourage participants to view difficulties as opportunities for growth rather than sources of stress. Under this approach, sports tourism can link short-term excitement with enduring well-being.

## 8 Limitations and future research

Despite its contributions, this study has several limitations that necessitate caution in generalizing the findings. First, the cross-sectional design does not allow strong causal claims; although the proposed directions of influence are theoretically grounded, they cannot be firmly established. Future research could employ longitudinal or experimental designs to track how situational interest and well-being evolve, offering deeper insights into their mutual influence. Second, reliance on self-reported data raises the possibility of common method bias. Adding observational, physiological, or qualitative measures could enrich the understanding of the psychological mechanisms involved. Third, while taking a multidimensional perspective on situational interest is a strength, future studies might investigate additional moderating variables—such as personality traits, social support, or cultural context—to clarify when challenge and exploration lead to or impede eudaimonic benefits.

Another promising direction involves examining transformational and sustainability-oriented sports tourism programs. Considering growing environmental concerns, research on how ecological awareness or nature-based activities interact with situational interest may yield meaningful insights for both theory and practice. Finally, expanding the scope to different sport types and cultural settings can test whether these findings hold universally or vary by destination, sport discipline, or demographic group. Addressing these issues will not only refine our understanding of how situational interest contributes to well-being but also help stakeholders design sports tourism experiences that are both meaningful and enduring.

## Data Availability

The original contributions presented in the study are included in the article/supplementary material, further inquiries can be directed to the corresponding author.
